# Multivariate Analysis of Photoacoustic Spectra for the Detection of Short-Chained Hydrocarbon Isotopologues

**DOI:** 10.3390/molecules25092266

**Published:** 2020-05-11

**Authors:** Alain Loh, Marcus Wolff

**Affiliations:** 1 Heinrich Blasius Institute of Physical Technologies, Hamburg University of Applied Sciences, Berliner Tor 21, 20099 Hamburg, Germany; marcus.wolff@haw-hamburg.de; 2 School of Computing, Engineering and Physical Sciences, University of the West of Scotland, High Street, Paisley PA1 2BE, UK

**Keywords:** multivariate analysis, partial least squares regression, photoacoustic spectroscopy, interband cascade laser, gas sensing, hydrocarbons, isotopologues, methane, ethane, propane

## Abstract

We report, to our knowledge, the first optical detection scheme for short-chained hydrocarbon isotopologues. The sensor system is based on photoacoustic spectroscopy (PAS). Two continuous wave, thermoelectrically cooled, distributed feedback interband cascade lasers (DFB-ICLs) with emission wavelengths around 3.33 and 3.38 μm, respectively, served as light sources. The investigations comprised the main stable carbon isotopologues of methane (^12^CH_4_, ^13^CH_4_), ethane (^12^CH_3_-^12^CH_3_, ^13^CH_3_-^12^CH_3_, ^13^CH_3_-^13^CH_3_), and propane (^12^CH_3_-^12^CH_2_-^12^CH_3_, ^13^CH_3_-^12^CH_2_-^12^CH_3_). They were selected because of their importance for numerous applications from climate and planetary research to natural gas exploration. Multiple measurements of single components in nitrogen and synthetic mixtures were conducted at room temperature and atmospheric pressure. Depending on the investigated hydrocarbon isotopologue, detection limits ranging from 0.043 ppmv to 3.4 ppmv were achieved. For a selective concentration determination, multivariate analysis (MVA) was applied. Partial least-squares regression (PLSR) was used to calculate concentrations from the PA spectra. The implementation of MVA has shown that the PA setup in principle works reliably and that the selective concentration determination of short-chained hydrocarbon isotopologues is possible.

## 1. Introduction

In environmental policy discussions, people often only talk about the carbon dioxide (CO_2_) emissions that need to be reduced and controlled in order to stop global warming. Due to simplified news coverage, many people are not aware that atmospheric methane (CH_4_) plays a major role in the greenhouse effect as it has a considerably higher global warming potential compared to CO_2_, i.e., CH_4_ emission will have 28 times the impact on temperature of a CO_2_ emission of the same mass over the following 100 years [1]. In addition to its own climate impact, the presence of CH_4_ in the atmosphere can also affect the concentration of other greenhouse gases, such as tropospheric ozone (O_3_), water vapor, and CO_2_ [2]. While CH_4_ does not cause direct harm to human health or crop production, its role as precursor gas contributes significantly to the negative health and agricultural impacts of O_3_ [3,4]. In contrast to CO_2_, the climate system reacts rapidly to changes in CH_4_ emissions and reducing CH_4_ emissions could provide the opportunity to immediately achieve major benefits for global and regional climate as well as for human health and agriculture [5]. A better understanding of the global methane budget is crucial to take meaningful measures. This can only be achieved by the sensitive measurement of short-chained (SC) hydrocarbons including their stable carbon isotopic ratios, as it can be used as a proxy for source and sink estimation (natural vs. anthropogenic), be an indicator for atmospheric processes, and therefore improve and correct atmospheric and climate models [6].

A second application involving hydrocarbon isotopologue detection concerns natural gas (NG) exploration. Natural gas is the fastest growing fossil fuel on the global energy market and consumption of NG worldwide is projected to increase more than 40% between 2018 and 2050. Thus, total consumption will reach nearly 200 quadrillion British thermal units (1 Btu = 1055 J) by 2050, according to the International Energy Outlook 2019 [7]. Asia especially will experience the largest rise in global NG consumption because the economies of big countries such as China and India are rapidly growing. Natural gas usage expedites the most to fulfil demand from increased industrial activity, NG-related electricity generation, and fuel-related transportation. The International Energy Outlook also stated that NG remains a key fuel in the electric power sector and in the industrial sector because in the power sector, NG is still an attractive choice for new generating plants due to its fuel efficiency. Natural gas burns cleaner than coal or oil products, and as more governments begin to implement national or regional plans to reduce CO_2_ emissions, they may promote the use of NG to replace more carbon-intensive coal and liquid fuels. Again, the sensitive measurement of the stable carbon isotopic composition of NG plays a key role in the production of this energy source. The ^13^C/^12^C ratio of SC hydrocarbons is strongly related to the gas type or source (i.e., bacterial, thermogenic, or mixed) and to its maturity [8]. A steadily growing share of NG production originates from shale. Several studies showed that high-production wells are often found in regions where a so called “isotopic reversal” in shale gas occurs [9,10,11]. The isotopic analysis in combination with conventional logging protocols can represent a powerful tool to control horizontal drilling and can significantly improve exploration, completion, and production technologies [12]. Ultimately, this will have both positive economic and ecological effects.

The standard technique for the measurement of carbon isotopes for the past 40 years has been the isotope ratio mass spectrometry (IRMS) together with the gas chromatography (GC) [13]. This method is highly precise but also labor-intensive, quite expensive, and typically performed in a facility, making real-time in-situ measurements impossible. GC-IRMS is also an indirect method and destroys the sample because the hydrocarbon must be converted into CO_2_ by burning prior to analyses of the isotope ratio. Further development of alternative isotope ratio measurement techniques is thus highly desirable. An alternative measurement method can be based on spectroscopy. The mid infrared (IR) range has proven to be particularly suitable for hydrocarbon analysis, since many hydrocarbon molecules exhibit strong rotational vibration transitions that are characteristic of the respective molecule. In the mid IR region, several laser sources are available, which include CO and CO_2_ gas lasers, lead-salt diode lasers, coherent sources based on difference frequency generation, optical parametric oscillators, quantum cascade lasers, and interband cascade lasers (ICLs) [14]. Distributed feedback (DFB) ICLs combine a good beam quality, single-mode operation, a small linewidth (<10 MHz) and continuous wave (cw) emission and mode-hop-free spectral tunability with a compact size, operation at room temperature and easy handling [15]. Together with their recent availability with wavelengths between 3 μm and 4 μm this makes them an excellent choice for the sensitive detection of SC hydrocarbon gases.

In a previous study, IR absorption cross sections of the ethane and propane isotopologues were measured for the first time in the mid IR range using an FTIR spectrometer [16]. Subsequently, high-resolution absorption cross sections of the same isotopologues were photo-acoustically measured using ICLs near 3.33 and 3.38 µm, respectively [15].

Photoacoustic spectroscopy (PAS) is based on the conversion of light to sound [17] and among spectroscopic techniques it offers many advantageous properties such as wide dynamic range, offset freeness, and simplicity [15]. It has been successfully applied in numerous trace gas sensing applications, which include atmospheric investigations (e.g., environmental monitoring, industrial emission measurements), detection of toxic gases, chemical warfare agents and explosives, biological and agricultural applications, industrial/manufacturing processes, and medical diagnostic (e.g., non-invasive breath analysis) [18,19,20].

In this work, we report the first PAS sensor for the detection of methane (CH_4_), ethane (C_2_H_6_), and propane (C_3_H_8_) including their main stable ^13^C-isotopologues (^13^CH_4_, ^13^CH_3_-^12^CH_3_, ^13^CH_3_-^13^CH_3_ and ^13^CH_3_-^12^CH_2_-^12^CH_3_) by using two DFB-ICLs operating in the wavelength range from 3327.7 nm to 3334.7 nm (ICL 1541, methane and ethane detection) and from 3373.3 nm to 3396.7 nm (ICL 1638, propane detection), respectively. In order to obtain the concentrations of individual substances of a measured spectrum containing multiple substances, multivariate analysis (MVA) [21,22] can be applied. This was also demonstrated in a recent work, where PA measurements of volatile organic compounds from strongly overlapping spectra in the mid IR region were evaluated [23]. Well-known multivariate regression models are the multiple linear regression, the principal component regression, and the partial least-square regression (PLSR), whereby the latter has become the standard method for spectroscopy [21,24]. If the data are noisy and strongly correlated, PLSR is considered to be superior to the other techniques [23,24]. Here, a PLSR model was trained and verified with experimental data of single and multiple components in synthetic gas mixtures. In addition, the sensor’s limit of detection (LOD) was determined.

## 2. Materials and Methods

### 2.1. Photoacoustic Spectroscopy

The PAS technique uses the fact that absorbed electromagnetic radiation is partially converted into kinetic energy by inelastic molecular collisions. This corresponds to an increase in the temperature of the irradiated volume. If the radiation is modulated, this will lead to modulated heating and, thus, generate a pressure wave with an amplitude proportional to the concentration of the absorbing molecules, which can be recorded with a microphone. For a single absorbing compound and under the condition that the absorption is not saturated, the wavelength-dependent PA signal S (in V) as function of the wavelength λ, is given as [15,25]

(1) $$S\left(\lambda \right)=CP\left(\lambda \right)N_{\mathrm{tot}}c\sigma \left(\lambda \right)$$

where C (in V/(cm^−1^ W)) is known as the cell constant depending mainly on the cell geometry but also on the cell wall surface, beam profile, the modulation frequency, and the microphone sensitivity. P(λ) (in W) is the laser power, $N_{\mathrm{tot}}$ is the total number density of molecules (in molecule/cm^3^), and the coefficients c and σ(λ) are the concentration and absorption cross section (in cm^2^ molecule^−1^) of the substance of interest, respectively. The PA signal is directly proportional to the laser power, concentration of the absorbing molecules, and the cell constant. If the laser modulation frequency corresponds to an acoustical resonance frequency of the cell, a significant signal amplification can be obtained.

### 2.2. Experimental Setup

**Figure 1:**
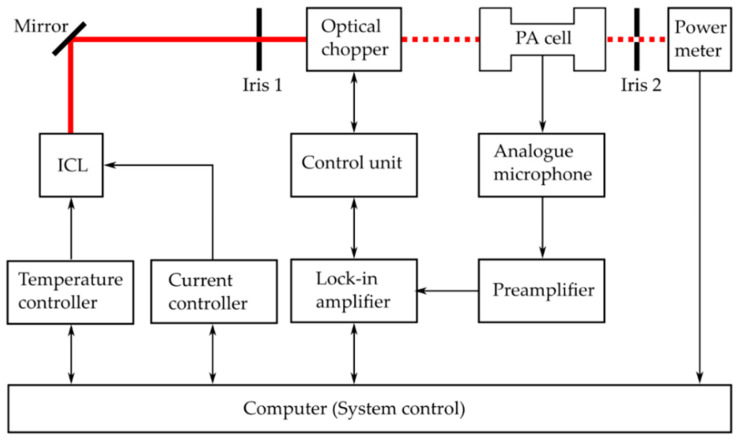
Experimental photoacoustic (PA) setup (block diagram).

A schematic diagram of the experimental setup is illustrated in Figure 1.

Two cw DFB-ICLs operating at wavelengths around 3.33 μm (ICL 1541, Nanoplus, Gerbrunn, Germany) and 3.38 μm (ICL 1638, Nanoplus), respectively, were selected as radiation sources for the isotope-selective detection of methane/ethane and propane. The laser sources (ICLs) were employed one after another. Each of them was operated by the same temperature controller (TTC001, Thorlabs, Newton, NJ, United States) and current controller (TLD001, Thorlabs). The laser beam modulated by an optical chopper (model 300CD, Scitec Instruments, Trowbridge, United Kingdom) and passes the H-shaped PA cell [26] before hitting a power meter (thermal head 3A-FS-SH, Ophir Optronics, Jerusalem, Israel). The power meter records the transmission spectrum. The linear response of the PA signal as function of laser power according to Equation (1) is in the strict sense only valid for weak absorptions. Stronger ones weaken the PA spectrum’s amplitudes and require a further correction. The spectral regions that required correction were determined by examining the transmission spectra. A mirror and two irises are required for the laser alignment. The chopper frequency at 2750 Hz is set by a dual phase digital signal processing lock-in amplifier (LIA: model 7265, Signal Recovery, Oak Ridge, TN, United States). The LIA with a time constant set to 1 s is also responsible for the signal processing of the preamplified (PAS PMV 201, PAS-Tech, Hamburg, Germany) PA signal recorded by the analogue microphone (EM-158N, Primo, Tokyo, Japan). The system control and the data acquisition are performed with a computer. Further details of the ICLs can be found elsewhere [15].

**Figure 2:**
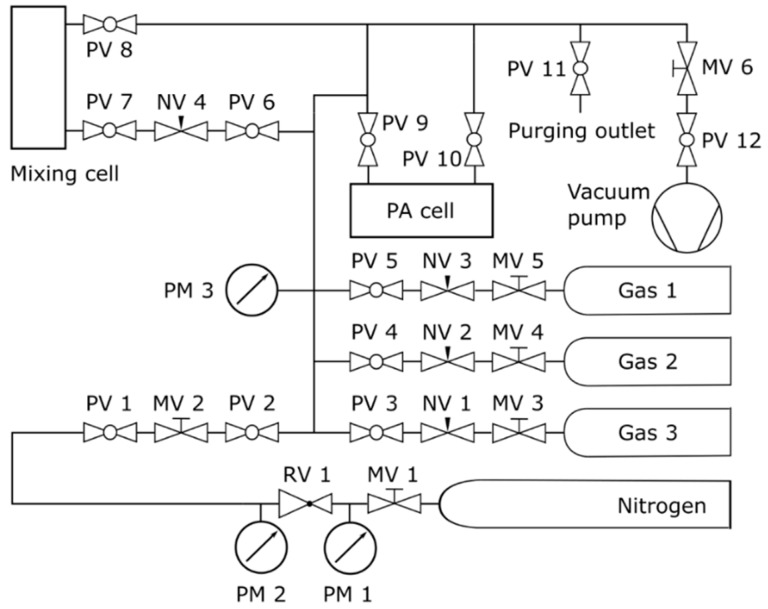
Photoacoustic spectroscopy (PAS) gas flow system. MVs are metering valves, PMs are pressure meters, RV is a pressure reducing valve, PVs are plug valves, and NVs are needle valves. The metering valves MV 1 and MV 3–5 are part of the pressure reducers. Pressure meters PM 1–2 and the reducing valve RV 1 belong to the N_2_ gas cylinder’s pressure reducer. The plug valves PV 1–6 separate the individual gas lines and PV 7–10 seal the mixing and the PA cell, respectively. The MV 2 and needle valves NV 1–4 allow a gentle filling of the cells. The MV 6 allows the adjustment of the vacuum pump power and the PV 11 and PV 12 close/open the purging outlet and the vacuum pump, respectively.

An overview of the PAS gas flow system is illustrated in Figure 2. 

Four gas lines, one for nitrogen (N_2_) and three for sample gases, lead to a connecting tube, which is attached to a pressure meter, the inlet of the mixing cell (for preprocessing) and to the PA cell. The gas lines consist partly of polytetrafluoroethylene and partly of stainless-steel tubes. The cell pressure was measured using a ceramic pressure transducer (PM 3) connected to the vacuum gauge (DVR 5, Vacuubrand, Wertheim, Germany). The transducer exhibits a resolution of 0.1 hPa in the range between 0.1 hPa and 10 hPa and a resolution of 1 hPa for pressures higher than 10 hPa. The outlets of the cells were connected to the vacuum pump, a chemistry-hybrid pump (RC 6, Vacuubrand), which enabled the evacuation of the gas lines including the cells down to 2.0 hPa. The sample mixtures were produced injecting a defined amount of one or more sample gases into the evacuated mixing cell and then adding N_2_ up to atmospheric pressure. Thereafter, the evacuated PA cell was filled with a small amount of the gas mixture from the mixing cell and, if required, additional amounts of different sample gases. Finally, the PA cell was filled with N_2_ up to atmospheric pressure to obtain the desired gas mixture. For some target concentrations, the usage of the mixing cell is not necessary. The concentrations, purities, and suppliers of the sample gases are listed in Table 1. The gases CH_4_, C_2_H_6_, and C_3_H_8_ were each present at natural abundance (NA).

Assuming that isotopes are randomly distributed throughout all isotopologues, the NA of the main methane isotopologues ^12^CH_4_ and ^13^CH_4_ are 98.8% and 1.11%, respectively [27]. The NA of the main ethane and propane isotopologues was estimated (based only on the ^13^C/^12^C ratio) to be ^12^CH_3_-^12^CH_3_: 97.8% ^13^CH_3_-^12^CH_3_: 2.2%, ^13^CH_3_-^13^CH_3_: 0.013%, ^12^CH_3_-^12^CH_2_-^12^CH_3_: 96.7%, and ^13^CH_3_-^12^CH_2_-^12^CH_3_: 2.2%, respectively.

### 2.3. Measurement and Data Processing

**Figure A1:**
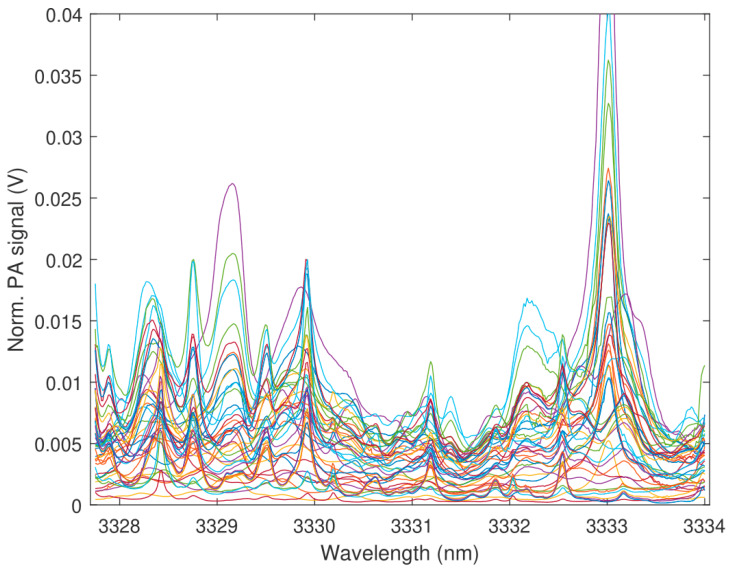
Measured PA spectra (43 different measurements) for multicomponent analysis with ICL 1541.

**Figure A2:**
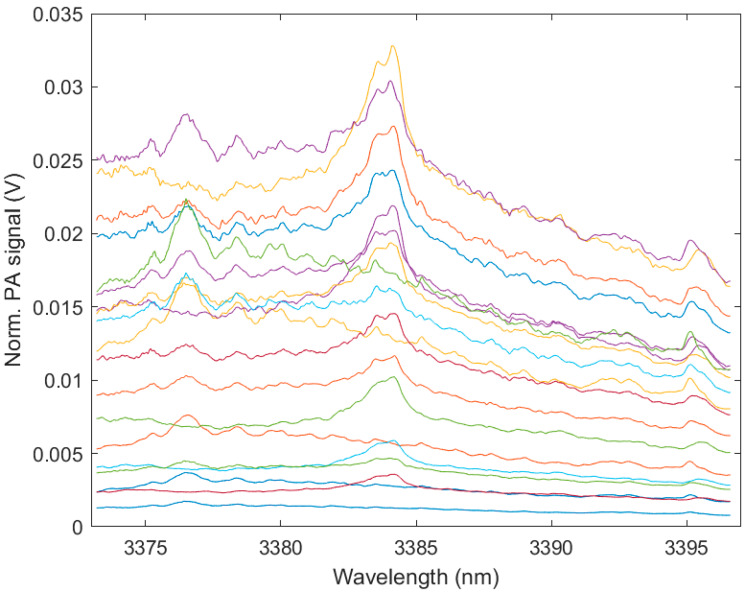
Measured PA spectra (19 different measurements) for multicomponent analysis with ICL 1638.

Measurements were performed on single and multiple component mixtures of sample gas with N_2_ under ambient conditions. In total, 62 (43 with ICL 1541 and 19 with ICL 1638) different SC hydrocarbon measurements/spectra were recorded. It should be noted, that a considerably larger dataset is usually required to set up a reliable prediction model. However, the primary goal of this research project was to show the feasibility. The ICL 1541 was used for all methane and ethane measurements, and the ICL 1638 for all propane measurements. The concentrations of the hydrocarbon used vary between 50 ppmv and 1 vol%. The individual concentrations for each measurement are listed in Table A1 of the Appendix A. The measured spectra are displayed in Figure A1 and Figure A2 of the Appendix A to give an impression of the difference.

Further details of the recording of the PA spectra with the ICLs and the processing of the spectra can be found elsewhere [15]. The processing involved averaging, power normalization, and wavelength correction.

### 2.4. Detection Limits

The signal-to-noise ratio (SNR) is commonly defined as the ratio of the signal power to the background noise power and is often expressed in dB. In this work, the SNR is calculated as the highest possible PA signal $S_{\max}$ of a specific substance divided by the standard deviation of the PA signal during a nitrogen measurement $\sigma_{N_{2}}$:

(2) $$\mathrm{SNR}\;=\;\frac{S_{\max}}{\sigma_{N_{2}}}$$

where SNR is of dimension 1 and $\sigma_{N_{2}}$ can be considered the noise level. PAS is a background-free technique, and, in principle, no PA signal is generated in absence of an absorber. Nitrogen does not show any absorption in the mid IR and therefore an N_2_ spectrum is perfectly suited to measure the background noise. The mean value of the PA signal during a N_2_ measurement represents an offset, e.g., due to window absorptions, and does not influence the SNR. The LOD is specified as the lowest quantity/concentration of a substance that can be distinguished from the absence of that substance. Here, the SNR shall be used for the calculation of the LOD of a substance:

(3) $$\mathrm{LOD}\;=\;\frac{c}{\mathrm{SNR}}$$

where c is the concentration of the substance during the SNR measurement. The obtained LOD is idealized, because it would be hard to distinguish a meaningful signal from the noise level, if both are of the same magnitude. Therefore, the value represents only a rough estimate of the experimentally achievable LOD. In order to measure the noise level of the setup, the sample cell was flushed with pure N_2_ and measurements were conducted under ambient conditions with both ICLs. The standard deviations of the N_2_ spectrum are 1.1 × 10^−5^ V for the ICL 1541 and 7.9 × 10^−6^ V for the ICL 1638, respectively.

### 2.5. Partial Least Squares Regression

Multicomponent measurements were processed with a PLSR model in the numerical software Matlab to evaluate the performance of the PA setup in detecting SC hydrocarbon isotopologue concentrations. The implementation of the PLSR method in Matlab is based on the SIMPLS algorithm developed by De Jong in 1993 [28]. The Matlab PLSR calculation function requires the gas spectra, the corresponding gas concentrations, and the number of selected latent variables (LVs) as input data. As output values, loading and score matrix of X and Y, explained variance (in %) by the model by each LV, and the PLSR coefficients are computed. The PLSR coefficients are required to calculate target values (concentrations) out of initial data (spectra):

(4) $$Y_{\mathrm{res}}=\;X_{\mathrm{pre}}B_{\mathrm{PLSR}}\;+\;G$$

where $B_{\mathrm{PLSR}}$ is the PLSR coefficient matrix, $X_{\mathrm{pre}}$ is the predictor matrix (spectra), $Y_{\mathrm{res}}$ the response matrix (concentrations), and G the error matrix [23,24].

The predictive accuracy, i.e., the standard/mean error of a multivariate calibration model, is expressed as the root-mean-square error (RMSE). For that, the predicted residual error sum of squares (PRESS) is calculated following the equation [21]:

(5) $$\mathrm{PRESS}\;=\;\overset{n}{\underset{i\;\;=1}{\sum }}{\left(y_{i}-{\hat{y}}_{i}\right)}^{2}$$

where $y_{i}$ denotes the initial data (true concentrations), ${\hat{y}}_{i}$ the predicted values (calculated concentrations) by the model and n the number of probes. The RMSE is then determined as

(6) $$\mathrm{RMSE}\;=\;\sqrt{\frac{\mathrm{PRESS}}{n}}$$

## 3. Results

**Figure 3:**
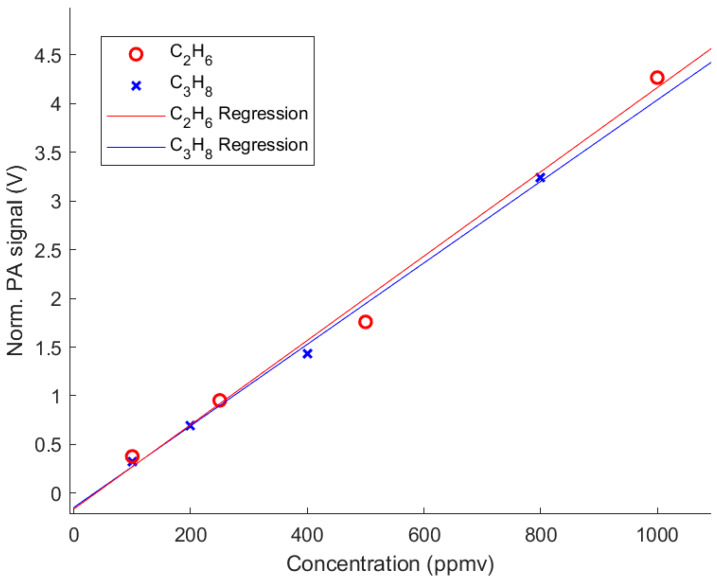
Linearity of the normalized PA signal (integrated over the whole spectrum) obtained of individual C_2_H_6_ and C_3_H_8_ measurements at different concentrations.

The linear behavior of the sensor was verified by evaluating the single-component gas measurements of the analytes. Representatively, the linearity of the single-component measurements of C_2_H_6_ and C_3_H_8_ are shown in Figure 3. The data demonstrate a good linearity with derived coefficients of determination of R^2^ = 0.991 (C_2_H_6_) and R^2^ = 0.997 (C_3_H_8_) and a small y-axis intercept of −0.162 V and −0.145 V, respectively. 

**Figure 4:**
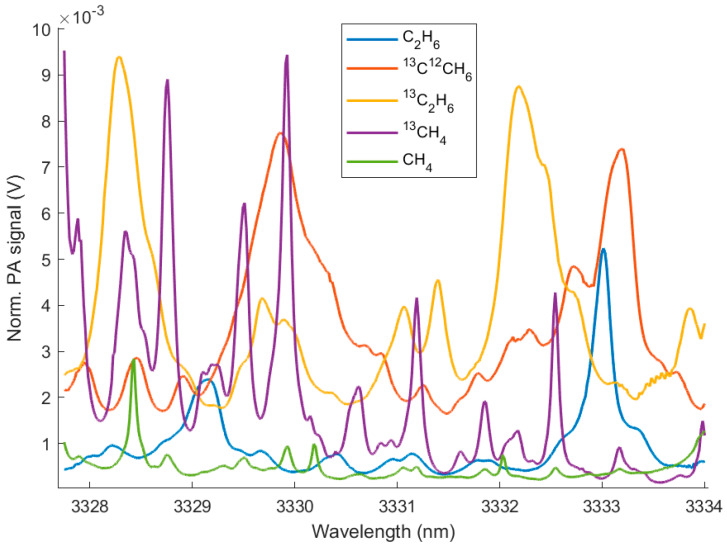
PA spectra measured with interband cascade lasers (ICL) 1541 of sample gases with concentrations according to Table 2.

**Figure 5:**
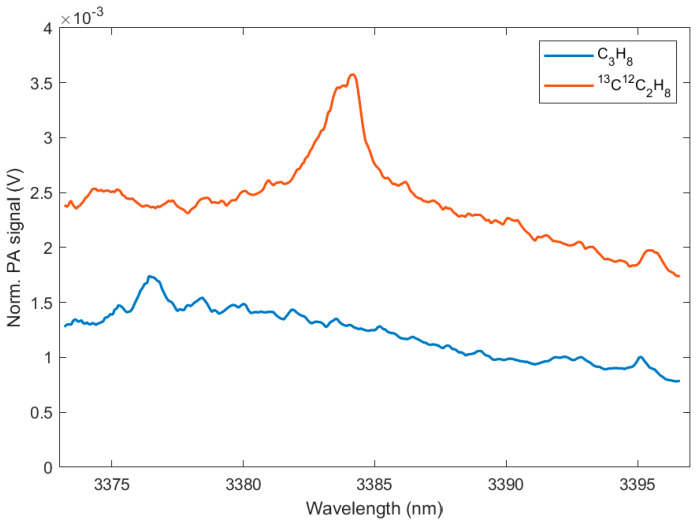
PA spectra measured with ICL 1638 of sample gases with concentrations according to Table 2.

The SNR and the LOD of the PAS sensor are calculated for each measured SC hydrocarbon isotopologue and are summarized in Table 2. In Figure 4, the respective PA spectra of the methane and the ethane isotopologues are shown. Although all these gases absorb across the entire wavelength range of the laser, each gas shows distinct absorption peaks, which makes them distinguishable from the others. In Figure 5, the PA spectra of the propane isotopologues are displayed. In contrast to the isotopologue spectra of methane and ethane, the propane isotopologue spectra show a broadband absorption structure with less sharp absorption peaks. In order to calculate the SNR, the maximum PA signal of each sample spectrum was determined from single component measurements with at low concentrations in N_2_. The SNR and LOD of the PAS sensor are different for each individual species. The PA signal depends equally on the absorption of the sample and on the laser power. This explains the noticeably higher LOD of CH_4_, because CH_4_ has a lower absorption strength (approximately one magnitude) in the spectral region of the laser source compared to the other compounds. It should be noted that due to the varying power performance of the ICLs at different wavelengths, the highest absorption peaks do not necessarily result in the maximum PA signal.

The method of choice for the validation of the PLSR model was a full cross validation (CV). Usually, all measurements have to be divided into calibration measurements to train the PLSR model (as a reference) and test measurements to verify the validity of the model. The CV is one of the most frequently used and efficient validation methods because it uses all recorded data for calibration as well as for validation [21]. In the full CV, n calibration models are created, where *n* stands for the number of probes (spectra). Each model uses n−1 probes for calibration and the left-out probe for validation. This is performed n times so that each probe serves once as validation. As part of the calibration, the PLSR model calculated five and two LVs for the measurements with the ICL 1541 and the ICL 1638, respectively, each LV representing the contribution of one hydrocarbon isotopologue. The 43/19 models were able to explain on average (96.7 ± 0.4)% (ICL 1541) and (99.1 ± 0.1)% (ICL 1638) of the total variance in the dataset regarding the target variable (**Y**).

**Figure 6:**
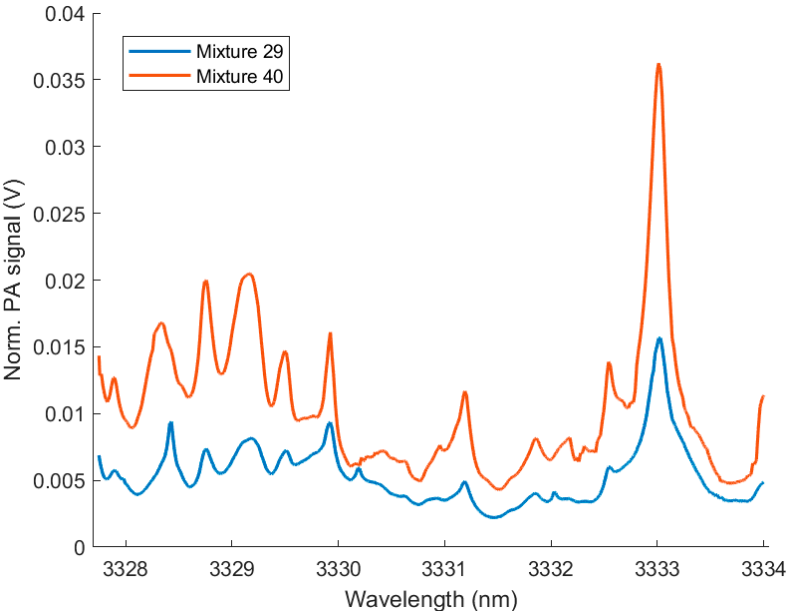
PA spectra measured with ICL 1541 of two multi-component mixtures with concentrations according to Table 3.

Figure 6 shows two exemplary PA measurements of multi-component mixtures. Table 3 lists the five concentrations determined by the pressure readings and the PLRS models.

The standard approach to evaluate the prediction/quality of the model would be to specify the error (RMSE) according to Equations (5) and (6) of each individual calibration model and average it over all RMSE in the course of the CV (RMSECV). The RMSEs of Mixtures 29 and 40 equal 123 ppmv and 562 ppmv, respectively. If we leave CH_4_ due to its comparably high concentration out, we get an RMSE of Mixture 29 of 13 ppmv and an RMSE of Mixture 40 of 98 ppmv. Hence, we consider a separation of the error into the respective components to be more informative, since, for example, larger concentrations were selected for CH_4_ (with possible larger errors) in order to obtain PA signals of the same magnitude as the other components. However, it is also possible for the PLSR to predict negative concentrations. Real concentrations are logically greater or equal to zero. Here, the negative predicted concentrations were set to zero. That way, the error (RMSECV_0_) could be slightly improved. In addition, in order to obtain a better understanding of the error in relation to the respective concentration, the CV mean relative residual (MRR) of the measurements of the individual components was determined. All the CV results are summarized in Table 4. 

**Figure A3:**
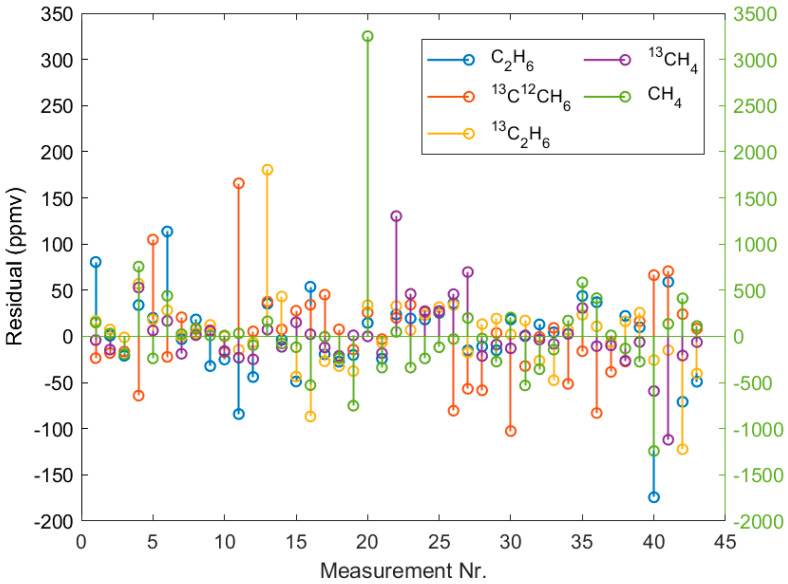
Cross validation residual of the ICL 1541 measurements. The y-axis on the right side refers to CH_4_ (green).

**Figure A4:**
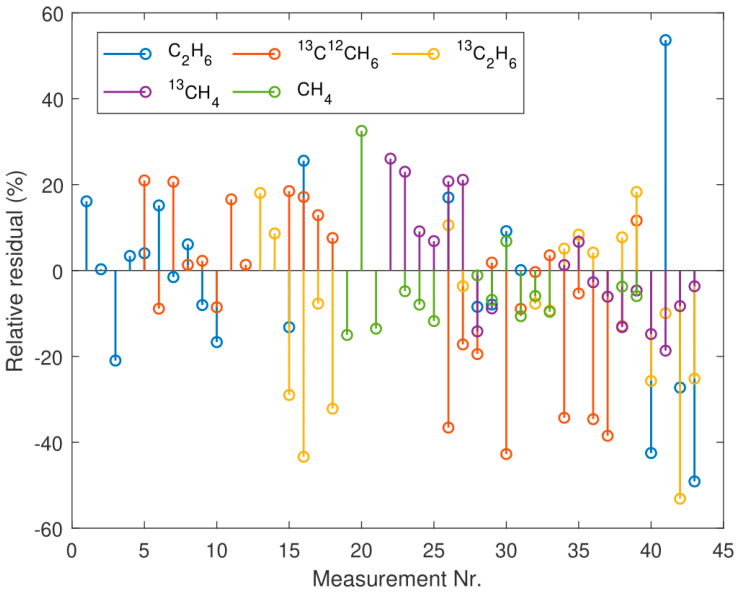
Cross validation relative residual of the ICL 1541 measurements.

**Figure A5:**
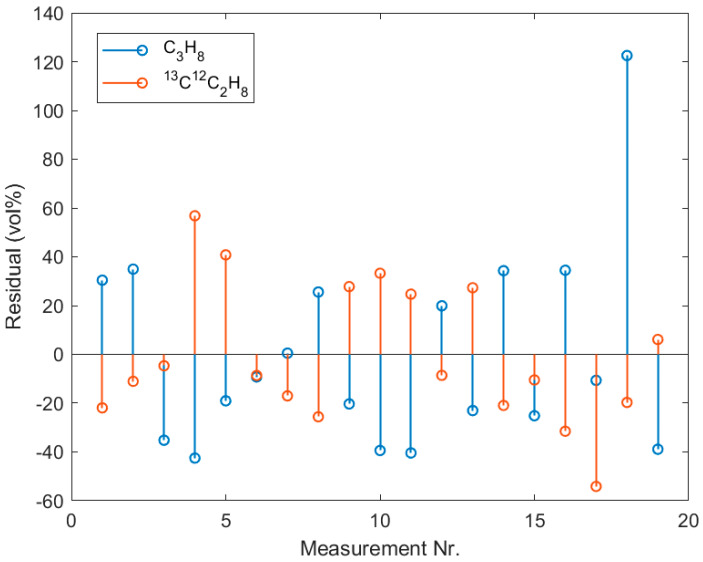
Cross validation residual of the ICL 1638 measurements.

**Figure A6:**
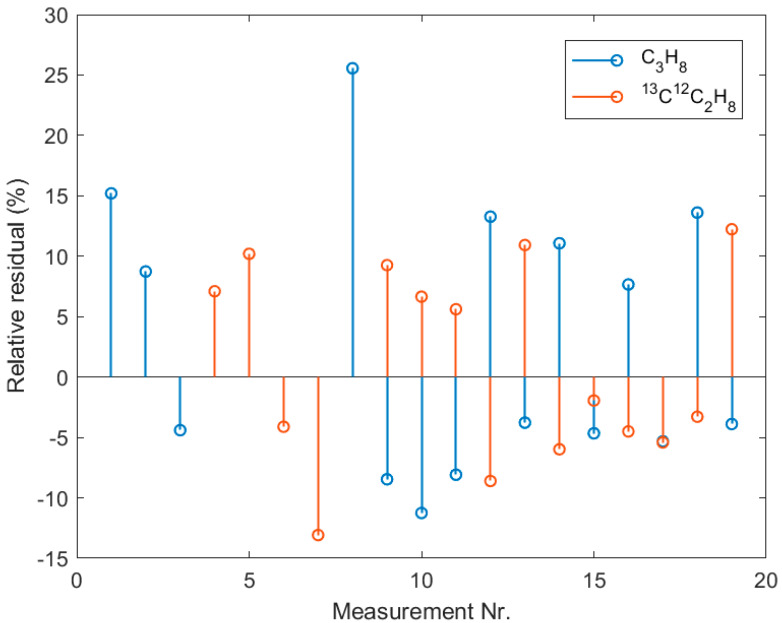
Cross validation relative residual of the ICL 1638 measurements.

The concentrations retrieved by the PLRS models can be obtained from Table A2 of the Appendix A. The residuals and the relative residuals of all predicted concentrations in the respective calibration model can be seen in Figure A3, Figure A4, Figure A5 and Figure A6 of the Appendix A, respectively. 

## 4. Discussion

In general, the quality of the MVA strongly depends on the applied calibration method as well as on the used spectra. The PLSR is applicable as long as the individual components contributing to the overall spectrum are linearly independent of each other and possess characteristic features with signals larger than the noise level. The recorded spectra depend on the experimental setup, the absorption strength of the investigated compounds, as well as their concentration. The smallest RMSECV (28 ppmv) and MRR (7.3%) were found for ^13^C^12^C_2_H_8_ measured with the ICL 1638. For the ICL 1541, the largest RMSECV (604 ppmv) and at the same time smallest MRR (9.7%) can be assigned to CH_4_. This proves that the RMSE is highly dependent on the concentration, which in the case of CH_4_ is, on average, one magnitude larger than the other hydrocarbons. The largest MMRs (15.6–17.6%) were found for the ethane isotopologues measured with the ICL 1541. These discrepancies between the expected and the regressed concentrations are too high to be attributed only to the regression algorithm. Moreover, the estimated errors for some concentration values such as the CH_4_ in Mixture 29 or the C_2_H_6_ in Mixture 40 appear to be too small to justify the difference with the retrieved concentration values. Additionally, the variation for retrieved concentrations where a gas component was absent is relatively large. For CH_4_, it varies between −756.2 ppmv in Mixture 4 and +1241 ppmv in Mixture 40. All these results point out an underlying problem with the gas line management and/or the recorded mixed sample spectra. Especially with a limited dataset, each single measurement has a significant influence on the model. The uncertainty of the results originates (in the sense of error propagation) from the uncertainty in the sample preparation as well as from all other influences of the experimental setup. The sample preparation is associated with a certain degree of inaccuracy. The method of preparing the gas mixtures results in an uncertainty of the partial pressures and, therefore, the concentrations. After inserting sample gas into the cell and closing the valves, the gas line between the cross-adaptor and the cell was still filled with this component. When the cell was subsequently filled up to ambient pressure with N_2_, this remainder was pushed into the cell, thus falsifying the sample gas concentration. In addition, the uncertainty in the sample preparation of multiple components is considerably higher because the determination is solely calculated out of the pressure readings of the gas system. Furthermore, it can be assumed that the different components are not particularly well mixed in the gas system and mixing cell and that unwanted remaining substances in the system cannot be completely excluded. In fact, the different components are linearly dependent of each other because all gases at NA include small proportions of their ^13^C isotopologues, which also increases the error. Considering the fact that in this work only a simple PA setup was realized without substantial efforts to maximize the SNR or LOD, these results are very promising, especially since many improvements are still possible, e.g., cell optimization, signal enhancement with optimized amplifier and LIA parameters, and the application of direct modulation of the ICL instead of using the mechanical chopper. 

## 5. Conclusions

Overall, the PA setup has demonstrated to work dependable in distinguishing SC hydrocarbon isotopologues and, in combination with the evaluation algorithm, enables an isotope-selective concentration determination of SC hydrocarbons. The current way of sample preparation offers room for improvement to minimize the concentration uncertainty. This can be achieved, for instance, with a suitable gas mixer, a system based on the combination of pressure controller and flow controller, and/or with the acquisition of hydrocarbon gas samples with accurate premixed concentrations. Afterwards, a proper design of experiment should be used for hydrocarbon concentration mixture combinations. This will help to plan necessary future measurements with synthetic multicomponent gas mixtures for the calibration model (required for the sensor’s concentration determination). In the future, the research will focus on several tasks concerning the optimization of the PA setup. The achievable LOD can be further enhanced by numerous experimental improvements. The used PA cell in this work has a relatively low Q-factor. An exchange of the current cell with an optimized PA cell with a higher Q-factor directly improves the PA signal and lowers the LOD. The feasibility of using a quartz tuning fork (QTF) as acoustic transducer will be further investigated, since QTFs can have Q values > 10,000 [29], which is unreachable with traditional PA cells. It should be noted that a reorientation to the quartz enhanced photoacoustic spectroscopy (QEPAS) technique (utilizing QTFs) is associated with several substantial changes of the PA setup. Commercially available QTFs work with a resonant frequency of ca. 32.8 kHz; the use of an optical chopper is therefore no longer possible. Therefore, the direct modulation of the ICL will be studied more intensively together with certain modulation methods like the wavelength modulation spectroscopy (WMS). Both changes offer the distinct advantage of efficient noise suppression [30,31]. In addition, the enhanced frequency stability of an applied direct modulation compared to an optical chopper together with the elimination of mechanical noise can result in better sensor sensitivity. The WMS approach was shown in a recent study using an ICL for the simultaneous detection of CH_4_, C_2_H_6_, and C_3_H_8_ (each at NA) using QEPAS [32]. In addition, the development of QEPAS is also still ongoing; therefore, further improvements will be achieved by implementing new generations of QTFs, overtone modes operation, and new micro-resonator systems [32].

## Figures and Tables

**Table 1:** Sample gases used in this work.

Sample Gas	Concentration	Purity	Supplier
CH_4_ (at NA)	1 vol%	3.5	Air Liquide S.A., Paris, France
^13^CH_4_	pure	2.0	Sigma-Aldrich, Inc., St. Louis, MO, United States
C_2_H_6_ (at NA)	100 ppmv	6.0	Linde AG., Dublin, Ireland
	1 vol%	3.5	Air Liquide S.A.
^13^C^12^CH_6_	pure	2.0	Sigma-Aldrich, Inc.
^13^C_2_H_6_	pure	2.0	Sigma-Aldrich, Inc.
C_3_H_8_ (at NA)	100 ppmv	6.0	Linde AG.
	1 vol%	3.5	Air Liquide S.A.
^13^C^12^C_2_H_8_	pure	2.0	Cambridge Isotope Laboratories, Inc. Tewksbury, MA, United States
N_2_	pure	5.0	Linde AG.

**Table 2:** Idealized detection limits of the PA setup. The signal-to-noise ratio (SNR) of each sample spectrum was obtained at these wavelengths.

Sample Gas	Concentration (ppmv)	Wavelength (nm)	SNR	LOD (ppmv)
CH_4_ (at NA)	2500	3328.4	728.9	3.4
^13^CH_4_	500	3329.9	4292.5	0.12
C_2_H_6_ (at NA)	100	3333.0	1449.7	0.069
^13^C^12^CH_6_	400	3329.9	3515.0	0.11
^13^C_2_H_6_	500	3332.2	3968.5	0.13
C_3_H_8_ (at NA)	100	3381.9	1564.3	0.064
^13^C^12^C_2_H_8_	130	3382.2	3039.8	0.043

**Table 3:** Concentrations of two exemplary chosen mixtures determined by the PLRS models.

Sample Gas	Concentration of Mixture 29 (ppmv)	PLRS Concentration (ppmv)	Concentration of Mixture 40 (ppmv)	PLRS Concentration (ppmv)
CH_4_ (at NA)	4000 ± 16	4273	0	1241
^13^CH_4_	100 ± 21	108	400 ± 55	459
C_2_H_6_ (at NA)	190 ± 10	205	410 ± 11	584
^13^C^12^CH_6_	200 ± 32	196	0	−66
^13^C_2_H_6_	0	−19	100 ± 21	126

**Table 4:** Summary of the cross validation (CV) results.

Sample Gas	RMSECV (ppmv)	RMSECV_0_ (ppmv)	MRR (%)
CH_4_ (at NA)	604	572	9.7
^13^CH_4_	35	34	12.4
C_2_H_6_ (at NA)	46	44	17.3
^13^C^12^CH_6_	49	45	15.6
^13^C_2_H_6_	43	41	17.6
C_3_H_8_ (at NA)	40	40	9.7
^13^C^12^C_2_H_8_	28	28	7.3

## Appendix A

**Table A1:** Hydrocarbon concentrations of the gas mixtures in ppmv. The compounds CH_4_, C_2_H_6_, and C_3_H_8_ are at NA. The uncertainties of the values were calculated from the purity of the sample gas and the error of the pressure readings (±1 hPa) by error propagation.

	ICL 1541						ICL 1638	
No.	C_2_H_6_	^13^C^12^CH_6_	^13^C_2_H_6_	CH_4_	^13^CH_4_	No.	C_3_H_8_	^13^C^12^C_2_H_8_
1	500 ± 11	0	0	0	0	1	200 ± 10	0
2	250 ± 10	0	0	0	0	2	400 ± 11	0
3	100	0	0	0	0	3	800 ± 11	0
4	1000 ± 12	0	0	0	0	4	0	800 ± 100
5	500 ± 11	500 ± 66	0	0	0	5	0	400 ± 55
6	750 ± 11	250 ± 38	0	0	0	6	0	210 ± 34
7	200 ± 10	100 ± 21	0	0	0	7	0	130 ± 25
8	300 ± 10	600 ± 77	0	0	0	8	100	0
9	400 ± 11	300 ± 44	0	0	0	9	240 ± 10	300 ± 44
10	150 ± 10	200 ± 32	0	0	0	10	350 ± 11	500 ± 66
11	0	1000 ± 122	0	0	0	11	500 ± 11	440 ± 59
12	0	400 ± 55	0	0	0	12	150 ± 10	100 ± 21
13	0	0	1000 ± 122	0	0	13	610 ± 11	250 ± 38
14	0	0	500 ± 66	0	0	14	310 ± 10	350 ± 49
15	370 ± 11	150 ± 27	150 ± 27	0	0	15	540 ± 11	540 ± 70
16	210 ± 10	200 ± 32	200 ± 32	0	0	16	450 ± 11	700 ± 88
17	0	350 ± 49	350 ± 49	0	0	17	200 ± 10	100 ± 21
18	0	100 ± 21	100 ± 21	0	0	18	900 ± 11	600 ± 77
19	0	0	0	5000 ± 18	0	19	1000 ± 12	50 ± 16
20	0	0	0	10000	0			
21	0	0	0	2500 ± 14	0			
22	0	0	0	0	500 ± 66			
23	0	0	0	7000 ± 21	200 ± 32			
24	0	0	0	3000 ± 15	300 ± 44			
25	0	0	0	1000 ± 12	400 ± 55			
26	210 ± 10	220 ± 35	320 ± 46	0	220 ± 35			
27	0	330 ± 47	480 ± 64	0	330 ± 47			
28	130 ± 10	300 ± 44	0	2000 ± 13	150 ± 27			
29	190 ± 10	200 ± 32	0	4000 ± 16	100 ± 21			
30	200 ± 10	240 ± 37	0	3000 ± 15	0			
31	300 ± 10	360 ± 50	0	5000 ± 18	0			
32	0	170 ± 29	340 ± 48	6000 ± 19	0			
33	0	260 ± 39	510 ± 67	1500 ± 12	0			
34	0	150 ± 27	140 ± 26	0	230 ± 36			
35	0	300 ± 44	280 ± 41	0	460 ± 62			
36	0	240 ± 37	260 ± 39	0	390 ± 54			
37	0	100 ± 21	100 ± 21	0	160 ± 28			
38	0	210 ± 34	210 ± 34	3500 ± 15	200 ± 32			
39	0	140 ± 26	140 ± 26	4600 ± 17	130 ± 25			
40	410 ± 11	0	100 ± 21	0	400 ± 55			
41	110 ± 10	0	150 ± 27	0	600 ± 77			
42	260 ± 10	0	230 ± 36	0	250 ± 38			
43	100	0	160 ± 28	0	170 ± 29			

**Table A2:** Hydrocarbon concentrations of the gas mixtures in ppmv determined by the PLRS models.

	ICL 1541						ICL 1638	
No.	C_2_H_6_	^13^C^12^CH_6_	^13^C_2_H_6_	CH_4_	^13^CH_4_	No.	C_3_H_8_	^13^C^12^C_2_H_8_
1	419.4	23.6	−16.8	−148.6	4.0	1	169.6	21.9
2	249.3	18.1	−7.5	−30.9	14.4	2	365.1	11.0
3	121.0	16.4	1.1	193.0	19.5	3	835.2	4.7
4	966.1	64.2	−57.0	−756.2	−52.9	4	42.5	743.2
5	480.0	395.1	−18.2	238.4	−6.4	5	19.0	359.2
6	636.4	272.2	−28.3	−439.3	−16.7	6	9.3	218.6
7	203.1	79.3	0.4	−25.0	18.8	7	−0.5	147.0
8	281.7	592.1	−3.4	−88.5	−1.3	8	74.4	25.6
9	432.1	293.2	−12.4	−8.8	−5.7	9	260.3	272.2
10	175.0	217.1	0.3	−9.2	15.9	10	389.4	466.7
11	84.2	834.2	14.4	−31.8	23.1	11	540.4	415.3
12	44.0	394.7	6.4	95.5	24.9	12	130.1	108.6
13	−35.3	−37.6	819.5	−163.7	−7.3	13	633.0	222.7
14	3.6	−7.5	456.8	77.4	11.3	14	275.7	370.9
15	418.8	122.3	193.5	117.6	−15.2	15	565.1	550.5
16	156.3	165.7	286.8	528.0	−2.3	16	415.5	731.5
17	19.4	304.9	377.0	3.5	12.0	17	210.6	1054.1
18	27.5	92.4	132.2	236.4	21.1	18	777.5	619.7
19	20.2	14.3	37.6	5750.5	−1.1	19	1038.9	43.9
20	−14.5	−25.8	−33.8	6748.3	0.0			
21	24.2	3.0	6.5	2838.3	18.2			
22	−23.7	−20.0	−32.8	−49.5	369.7			
23	−19.4	−34.3	−6.8	7337.5	154.0			
24	−18.3	−26.2	−23.0	3238.5	272.7			
25	−25.4	−26.8	−31.7	1117.7	372.6			
26	174.3	300.5	286.1	27.9	174.3			
27	15.1	386.8	497.5	−199.5	260.3			
28	141.0	358.4	−13.2	2022.8	171.3			
29	205.1	196.3	−19.3	4273.1	108.8			
30	181.7	342.6	−2.1	2795.9	13.0			
31	299.7	392.2	−17.2	5531.2	−1.1			
32	−13.0	170.6	366.3	6355.6	3.6			
33	−4.5	250.8	557.5	1644.1	8.1			
34	−2.7	201.4	132.9	−171.1	227.1			
35	−43.8	316.0	256.6	−584.8	429.3			
36	−37.0	323.1	249.2	−413.9	400.6			
37	6.2	138.5	106.1	−10.9	169.8			
38	−22.2	237.3	193.8	3630.9	226.4			
39	−9.8	123.7	114.4	4874.5	136.0			
40	584.3	−66.4	125.7	1241.4	459.2			
41	51.0	−70.7	164.9	−135.6	712.0			
42	330.9	−24.1	352.2	−412.2	270.7			
43	149.1	−8.4	200.3	−112.3	176.2			

## References

[1] Myhre G., Shindell D., Bréon F.-M., Collins W., Fuglestvedt J., Huang J., Koch D., Lamarque J.-F., Lee D., Mendoza B., Contribution of Working Group I to the Fifth Assessment Report of the Intergovernmental Panel on Climate Change (2013). Anthropogenic and Natural Radiative Forcing. Climate Change 2013: The Physical Science Basis.

[2] Warneck P. (2000). Chemistry of the Natural Atmosphere.

[3] Chen T.-M., Kuschner W.G., Gokhale J., Shofer S. (2007). Outdoor air pollution: Ozone health effects. Am. J. Med. Sci..

[4] van Dingenen R., Dentener F., Raes F., Krol M., Emberson L., Cofala J. (2009). The global impact of ozone on agricultural crop yields under current and future air quality legislation. Atmos. Environ..

[5] Shindell D., Kuylenstierna J.C.I., Vignati E., Van Dingenen R., Amann M., Klimont Z., Anenberg S.C., Muller N., Janssens-Maenhout G., Raes F. (2012). Simultaneously mitigating near-term climate change and improving human health and food security. Science.

[6] Gensch I., Kiendler-Scharr A., Rudolph J. (2014). Isotope ratio studies of atmospheric organic compounds: Principles, methods, applications and potential. Int. J. Mass Spectrom..

[7] U.S. Energy Information Administration (2019). International Energy Outlook 2019: With Projections to 2050.

[8] Faramawy S., Zaki T., Sakr A.-E. (2016). Natural gas origin, composition, and processing: A review. J. Nat. Gas Sci. Eng..

[9] Zumberge J.E., Ferworn K., Brown S. (2012). Isotopic reversal (‘rollover’) in shale gases produced from the Mississippian Barnett and Fayetteville formations. Mar. Pet. Geol..

[10] Xia X., Chen J., Braun R., Tang Y. (2013). Isotopic reversals with respect to maturity trends due to mixing of primary and secondary products in source rocks. Chem. Geol..

[11] Tilley B., Muehlenbachs K. (2013). Isotope reversals and universal stages and trends of gas maturation in sealed, self-contained petroleum systems. Chem. Geol..

[12] Ellis L., Brown A., Schoell M., Uchytil S. (2003). Mud gas isotope logging (MGIL) assists in oil and gas drilling operations. Oil Gas J..

[13] Sessions A.L. (2006). Isotope-ratio detection for gas chromatography. J. Sep. Sci..

[14] Vurgaftman I., Weih R., Kamp M., Meyer J.R., Canedy C.L., Kim C.S., Kim M., Bewley W.W., Merritt C.D., Abell J. (2015). Interband cascade lasers. J. Phys. D Appl. Phys..

[15] Loh A., Wolff M. (2019). High resolution spectra of 13C ethane and propane isotopologues photoacoustically measured using interband cascade lasers near 3.33 and 3.38 µm, respectively. J. Quant. Spectrosc. Radiat. Transf..

[16] Loh A., Wolff M. (2017). Absorption cross sections of 13C ethane and propane isotopologues in the 3 µm region. J. Quant. Spectrosc. Radiat. Transf..

[17] Bell A.G. (1880). On the production and reproduction of sound by light. Am. J. Sci..

[18] Harren F.J.M., Christescu S.M., Meyers R.A. (2006). Photoacoustic Spectroscopy in Trace Gas Monitoring: (2019 version). Encyclopedia of Analytical Chemistry.

[19] Elia A., Lugarà P.M., Di Franco C., Spagnolo V. (2009). Photoacoustic techniques for trace gas sensing based on semiconductor laser sources. Sensors.

[20] Patimisco P., Scamarcio G., Tittel F.K., Spagnolo V. (2014). Quartz-enhanced photoacoustic spectroscopy: A review. Sensors.

[21] Kessler W. (2006). Multivariate Datenanalyse in der Bio- und Prozessanalytik: Mit Beispielen aus der Praxis.

[22] Hair J.F., Black W.C., Babin B.J., Anderson R.E. (2010). Multivariate Data Analysis.

[23] Saalberg Y., Wolff M. (2018). Multivariate Analysis as a Tool to Identify Concentrations from Strongly Overlapping Gas Spectra. Sensors.

[24] Wold S., Sjöström M., Eriksson L. (2001). PLS-regression: A basic tool of chemometrics. Chemom. Intell. Lab. Syst..

[25] Sigrist M.W. (1994). Laser photoacoustic spectrometry for trace gas monitoring. Analyst.

[26] Nodo E. (1978). Optimization of resonant cell design for optoacoustic gas spectroscopy (H-type). Appl. Opt..

[27] Stolper D., Sessions A.L., Ferreira A., Neto E.S., Schimmelmann A., Shusta S., Valentine D.L., Eiler J. (2014). Combined 13C–D and D–D clumping in methane: Methods and preliminary results. Geochim. Cosmochim. Acta.

[28] de Jong S. (1993). SIMPLS: An alternative approach to partial least squares regression. Chemom. Intell. Lab. Syst..

[29] Patimisco P., Sampaolo A., Dong L., Tittel F.K., Spagnolo V. (2018). Recent advances in quartz enhanced photoacoustic sensing. Appl. Phys. Rev..

[30] Schilt S., Thévenaz L. (2006). Wavelength modulation photoacoustic spectroscopy: Theoretical description and experimental results. Infrared Phys. Technol..

[31] Li J., Du Z., An Y. (2015). Frequency modulation characteristics for interband cascade lasers emitting at 3 μm. Appl. Phys. B.

[32] Sampaolo A., Csutak S., Patimisco P., Giglio M., Menduni G., Passaro V., Tittel F.K., Deffenbaugh M., Spagnolo V. (2019). Methane, ethane and propane detection using a compact quartz enhanced photoacoustic sensor and a single interband cascade laser. Sens. Actuators B Chem..

